# Analgesic effects of low-dose ketamine after spinal fusion in adults: A protocol of prospective randomized trial

**DOI:** 10.1097/MD.0000000000022162

**Published:** 2020-09-18

**Authors:** Hua Wang, Long Ma, Yongxue Chen

**Affiliations:** Department of Anesthesiology, Handan Central Hospital, Hebei Province, China.

**Keywords:** ketamine, pain management, random, spinal fusion, study protocol

## Abstract

**Background:**

Patients with spinal fusion often have opioid tolerance and chronic pain, which makes it difficult to control postoperative pain. In this double-blind, randomized, prospective study, we assessed the safety and efficacy of intravenous low-dose ketamine for the treatment of pain in patients undergoing the lumbar spinal fusion.

**Methods:**

This randomized, prospective, double-blind and placebo-controlled study was approved via the hospital institutional review committee. Patients were registered with signed written consent. All the floor nurses, recovery room and surgeons, patients, statisticians as well as research assistants were unaware of the grouping. The patients were randomly divided into ketamine group and control group by random number table. Nausea, vomiting or vomiting, the intensity of pain, adverse events, cumulative morphine consumption, as well as the amount of extra antiemetics or analgesics were evaluated at 6 hours, 12 hours, 24 hours, 36 hours, and 48 hours after the operation. *P* < .05 was considered to be the statistically significant. The Statistical Package for the software of Social Sciences 20.0 was utilized for statistical analysis.

**Conclusions:**

For the present trial, we assumed that intravenous ketamine could improve the satisfaction of patient by reducing the total consumption of morphine equivalent and the pain scores.

**Trial registration:**

This study protocol was registered in Research Registry (researchregistry5896).

## 1 Introduction

The management of postoperative pain in patients with chronic pain remains a huge clinical challenge. Patients with spinal fusion often have opioid tolerance and chronic pain, which makes it difficult to control postoperative pain.^[1]^ Because of the intractable pain caused by extensive injury of muscles, bones and soft tissue, sometimes pain tolerance is impossible, and the patient spent painful hours early after the operations. Postoperative pain may also affect other aspects of treatment such as restriction of limbs motility or ventilation.^[2–4]^

Ketamine is a kind of intravenous anesthetic agent and a noncompetitive blocker for the N-methyl-D-aspartate receptors.^[5]^ Ketamine has immunosuppressive effects, antioxidant, and anti-inflammatory. The action mechanism of ketamine is related to the binding of phencyclidine site to glutamate receptors.^[6]^ Glutamate mediation effect determine its neuroprotective, dissociative and analgesic effects.^[7]^ Ketamine is now extensively utilized as a non-opioid analgesic for spinal fusion, reducing the need for opioids. Despite the ketamines benefits, the use of ketamine can present major challenges, including delirium, which appears in almost 50% of patients after the ketamine administration.^[8]^

The clinical researches have indicated decreased opioid demands after operation and improved the pain control in patients with opioid tolerance with intra-operative injection of ketamine.^[9–15]^ Nevertheless, ketamine has not yet been authorized via the Food and Drug Administration to decrease the demands for opioids. Thus, the safety and effectiveness of low-doses ketamine in the spinal fusion need to be further investigated. In this double-blind, randomized, prospective study, we assessed the safety and efficacy of intravenous low-dose ketamine for the treatment of pain in patients undergoing the lumbar spinal fusion. Although ketamine is administered by different routes, the side effects can be better controlled by intravenous administration because of its lower half-life. We assumed that intravenous ketamine could improve the satisfaction of patient by reducing the total consumption of morphine equivalent and the pain scores.

## 2 Material and method

### 2.1 Study design and patient enrollment

This randomized, prospective, double-blind and placebo-controlled study was approved via the institutional review committee of Handan Central Hospital (HBHD70442). Prior to registration, this trial has been registered with the Research Registry (researchregistry5896). Patients were registered with signed written consent. All the floor nurses, recovery room and surgeons, patients, statisticians as well as research assistants were unaware of the grouping. Only the nurses in operating room were not blinded.

A prospective study were conducted in patients aged from 16 to 75 years and ASA physical condition 1 to 3 years who planned to undergo the elective lumbar fusion under the condition of general anesthesia. Exclusion criteria included poor control of hypertension, serious pulmonary or cardiac disease, severe renal or hepatic insufficiency, and elevated intraocular pressure, pregnancy, with the history of mental disorders, inability to operate patient-controlled analgesia pump, understand numerical pain scale, and speak Chinese, known allergies to hydromorphone or ketamine.

### 2.2 Randomization

**Figure 1:**
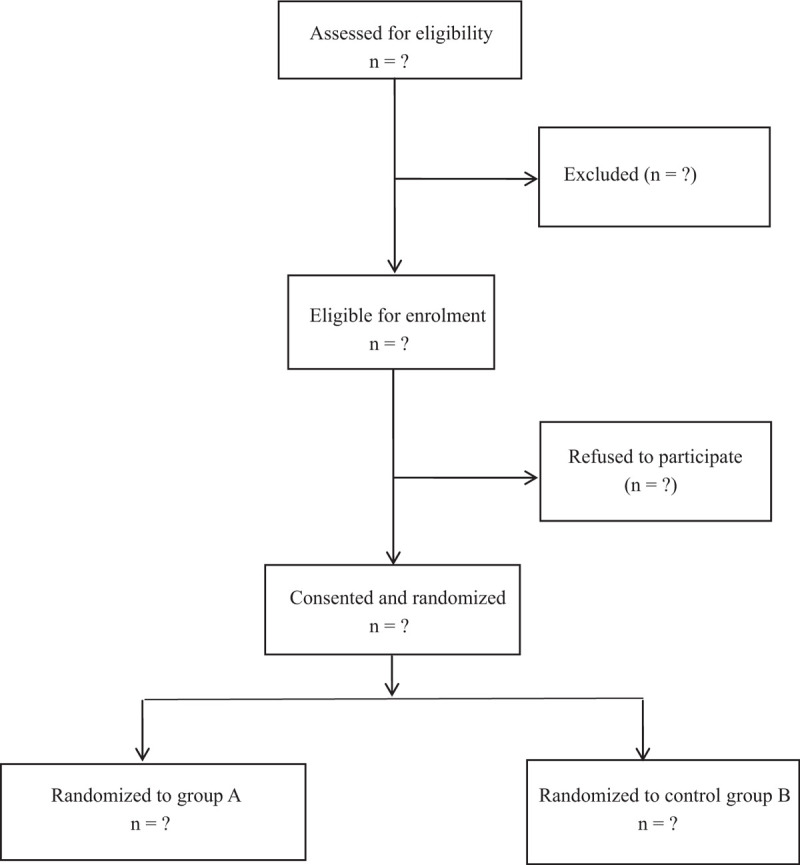
Consolidated Standards of Reporting Trials Statement flow diagram.

The patients were randomly divided into ketamine group and control group by random number table. And group assignment was assigned in opaque envelopes with sequential numbers. On the operation day, a nurse who was not involved in the evaluation or management of the patient opened the envelope and then prepared the ketamine or an equivalent amount of the normal saline as assigned. Before the end of this research, the assignments of group are not made public to researchers or patients (Fig. 1).

### 2.3 Anesthetic protocol

Each patient was given a standard protocol of general anesthesia. All the patients were induced by intravenous Target Controlled Infusion (TCI) 5 ng/ml of remifentanil and intravenous TCI 4 μg/ml of Propofol as well as intravenous 1 mg/kg of Rocuronium for the convenience of tracheal intubation. During the surgery, TCI 2–8 ng/ml of remifentanil and TCI 2.5–4 μg/ml of Propofol were utilized to maintain anesthesia, and the value of Bispectral Index was maintained at 40 to 60. Both the 2 groups were given 20 mg/kg of tranexamic acid intravenously before skin incision and after induction. If necessary, intravenous Rocuronium and the muscle relaxant were given throughout this procedure. The mean patient arterial pressure was kept above 60 mm Hg. The patient was kept at a normal temperature with 36 to 37°C core temperature. In the whole operation process, we utilized the water bath coaxial fluid and blood warming device and forced air warming device. All the cases were treated with the system of cell salvage autologous blood recovery.

### 2.4 Interventions

In the patients of Group A, blind test solution (0.05 ml/kg, namely, 0.5 mg/kg of the ketamine) was administered intravenously after orotracheal intubation and prior to skin incision for more than 2 minutes. After the initial administration, the minimum value of 3 μg/kg ketamine was maintained, this continues until patient recovered from the anesthesia. Afterward, the infusion rate decreased to 1.5 μg/kg.min and it kept for 2 days. Patients in Group B were received the same amount of normal saline.

### 2.5 Outcomes measures

Nausea, vomiting or vomiting, the intensity of pain, adverse events, cumulative morphine consumption, as well as the amount of extra antiemetics or analgesics were evaluated at 6 hours, 12 hours, 24 hours, 36 hours, and 48 hours after the operation. Patients needed to score their most severe rest pain and nausea at each time point. Pain during movement was evaluated when the patient tried to lift both legs in a supine position. Nausea was an unpleasant feeling related to the awareness of urge to vomit; and the vomiting represents the forced removal of the gastric contents from mouth. We recorded the absence/existence of the nausea and the number of vomiting episodes (over 10 ml).

### 2.6 Sample size calculation

The pre-study data of our department revealed that the average morphine consumption of patients with spinal fusion from 0 to 24 hours after the operation was 36 mg and standard deviation was 24. With the type 1 5% of error (α) and 80% of power (1 - β), the calculations of sample size indicated that each group of 60 patients needed to determine a reduction of 30% in the morphine consumption. Considering the uncertainty in our calculation of standard deviation and dropouts, we decided to include 150 subjects (75 in each group).

### 2.7 Statistical analysis

In this present study, the Statistical Package for the software of Social Sciences (SPSS) 20.0 was utilized for statistical analysis. The continuous variables were expressed with the form of error or mean ± standard deviation. And the continuous variables was evaluated with Kolmogorov-Smirnov normality test. Group comparisons on the variables that showed normal distribution were performed using one-way analysis of variance. The Mann-Whitney U analysis of variance was utilized for the discrete numerical variables that did not display the normal distribution. The chi-square (χ^2^) test and crosstabs were utilized to detect the relationship between categorical variables. And *P* < .05 was considered to be the statistically significant.

## 3 Discussion

Ketamine is commonly used in spine surgery as a nonopioid analgesic, decreasing opioid requirements. The beneficial and adverse effects of ketamine are dose-related, but optimal dosing of ketamine remains undetermined. In this double-blind, randomized, prospective study, we assessed the safety and efficacy of intravenous low-dose ketamine for the treatment of pain in patients undergoing the lumbar spinal fusion. We assumed that intravenous ketamine could improve the satisfaction of patient by reducing the total consumption of morphine equivalent and the pain scores.

## Author contributions

**Conceptualization:** Yongxue Chen.

**Data curation:** Hua Wang, Long Ma.

**Formal analysis:** Hua Wang, Long Ma.

**Funding acquisition:** Yongxue Chen.

**Investigation:** Hua Wang, Long Ma.

**Methodology:** Yongxue Chen.

**Resources:** Yongxue Chen.

**Software:** Yongxue Chen.

**Supervision:** Yongxue Chen.

**Validation:** Long Ma.

**Visualization:** Long Ma.

**Writing – original draft:** Hua Wang.

**Writing – review & editing:** Yongxue Chen.

## Abbreviations

Abbreviation: TCI = Target Controlled Infusion.
